# Direct phenotypic conversion of human fibroblasts into functional osteoblasts triggered by a blockade of the transforming growth factor-β signal

**DOI:** 10.1038/s41598-018-26745-2

**Published:** 2018-05-31

**Authors:** Kenta Yamamoto, Tsunao Kishida, Kei Nakai, Yoshiki Sato, Shin-ichiro Kotani, Yuta Nishizawa, Toshiro Yamamoto, Narisato Kanamura, Osam Mazda

**Affiliations:** 1 Department of Immunology, Kyoto Prefectural University of Medicine, Kamigyo, Kyoto 602-8566 Japan; 2 Department of Dental Medicine, Kyoto Prefectural University of Medicine, Kamigyo, Kyoto 602-8566 Japan

## Abstract

A procedure to generate functional osteoblasts from human somatic cells may pave the way to a novel and effective transplantation therapy in bone disorders. Here, we report that human fibroblasts were induced to show osteoblast phenotypes by culturing with ALK5 i II, which is a specific inhibitor for activin-like kinase 5 (ALK5) (tumor growth factor-β receptor 1 (TGF-β R1)). Cells cultured with ALK5 i II expressed osteoblast-specific genes and massively produced calcified bone matrix, similar to the osteoblasts induced from mesenchymal stem cells (MSC-OBs). Treatment with vitamin D3 in addition to ALK5 i II induced more osteoblast-like characters, and the efficiency of the conversion reached approximately 90%. The chemical compound-mediated directly converted osteoblasts (cOBs) were similar to human primary osteoblasts in terms of expression profiles of osteoblast-related genes. The cOBs abundantly produced bone matrix *in vivo* and facilitated bone healing after they were transplanted into immunodeficient mice at an artificially induced defect lesion in femoral bone. The present procedure realizes a highly efficient direct conversion of human fibroblasts into transgene-free and highly functional osteoblasts, which might be applied in a novel strategy of bone regeneration therapy in bone diseases.

## Introduction

Various bone diseases may cause serious and long-lasting locomotor disabilities that bring a substantial reduction in the quality of life and ability of daily living of the patients. They include non-union after osteoporotic bone fracture, large bone defects due to severe trauma, bone resorption due to multiple myeloma and osteolytic metastatic tumors, and bone destruction associated with progression of joint diseases such as rheumatoid arthritis and osteoarthritis. The osteoblasts play central roles in bone formation and remodeling by producing bone matrix^1^. Transplantation of osteoblasts into the bone lesions may greatly accelerate bone healing and boost functional recovery.

Recent advances in somatic cell reprogramming technologies have enabled direct conversion of somatic cells (such as fibroblasts) into cells of other lineages, including cardiomyocytes, neuronal cells, chondrocytes, hepatocytes, etc.^2–8^. We previously found that human fibroblasts were directly converted into osteoblasts at an efficiency of approximately 80% upon transducing Runx2, osterix, Oct3/4, and L-myc genes by means of the retroviral vectors^9^. Directly converted fibroblasts (dOBs) massively produced bone matrix, exhibited a genome-wide gene expression profile similar to human bone-derived osteoblasts, and promoted bone regeneration after being grafted into an artificially induced bone defect lesion in mice^9^. The advantage of this technology in regenerative therapy against bone diseases was that osteoblasts with high bone forming ability could be generated from fibroblasts that were collected from even elderly patients through a minimally invasive procedure. However, the use of retroviral vectors might result in the integration of exogenous gene sequences into the chromosomes, so that a small population of the transduced cells could potentially give rise to tumor cells after transplantation. To prevent such possible adverse events, we substituted a plasmid vector for the retroviral vectors. The osterix, Oct3/4 and L-myc genes were inserted into a single plasmid, which was then transfected into human fibroblasts by electroporation. In this way, we succeeded in inducing plasmid-driven directly converted osteoblasts (p-dOBs) with much less propensity to form tumors, because their chromosomes may remain intact^10^. If osteoblasts could be induced from fibroblasts without transferring any exogenous gene, such a procedure could produce osteoblasts that may be transplanted into patients with minimum risk of tumorigenesis.

It has been reported that an addition of some chemical compounds elevated the efficiency, and accelerated the processes, of generation of induced pluripotent stem (iPS) cells from somatic cells transduced with Yamanaka’s factor genes. These include histone deacetylase inhibitors^11^, DNA methyltransferase^11^, TGF-β receptor (TGF-β R) inhibitors^12–14^, a MEK-ERK pathway inhibitor^12,13,15^, a GSK3 inhibitor^15^ and arginine methyltransferases^16^. Some compounds including a histone deacetylase inhibitor^17^, TGF-β R inhibitors^18^, and a GSK3 inhibitor^19,20^ were capable of replacing one or more Yamanaka’s factor(s) in inducing somatic cell reprogramming. More recent studies demonstrated that fibroblasts were directly converted into neuronal cells^21,22^ and cardiomyocytes^23^ by culturing the cells with particular cocktails of chemical compounds. In this context, we tried to establish a procedure that realizes chemical compound(s)-mediated conversion of human fibroblasts into osteoblasts.

## Results

### Human dermal fibroblasts (HDFs) were induced to show osteoblast-like phenotype by treatment with ALK5 i II

**Figure 1:**
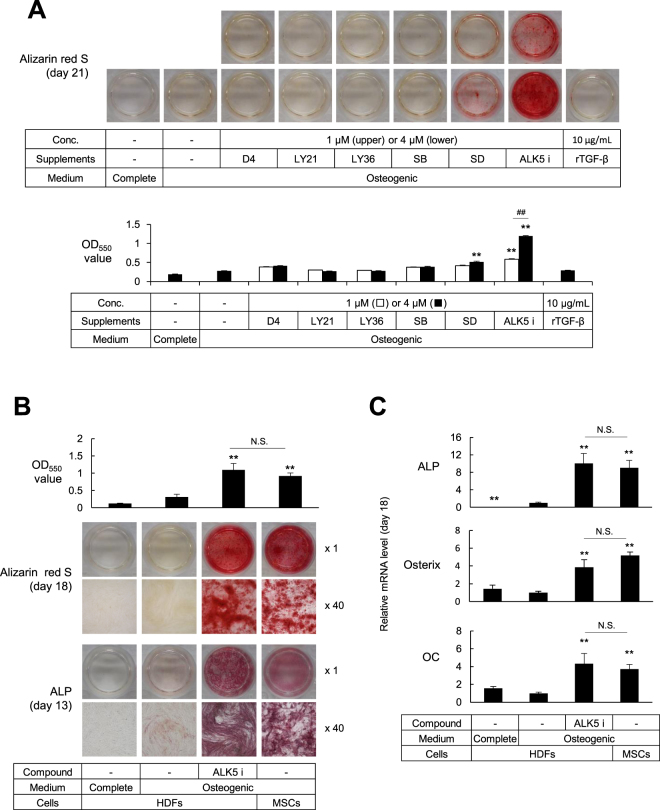
Osteoblast-like phenotypes were induced in human fibroblasts cultured in osteogenic medium supplemented with ALK5 i II. (**A**) Human dermal fibroblasts (aHDFs) were seeded into 24-well plates and cultured in the complete medium or osteogenic medium supplemented with TGF-β receptor inhibitors or rTGF-β as indicated. After culturing for 21 days, cells were stained with Alizarin Red S. Macroscopic images (Upper) and staining intensities (OD_550_) (Lower) are shown. (**B**) aHDFs and MSCs were seeded into 24-well plates (day 0) and cultured as in (**A**). Thirteen days later, cells were subjected to ALP staining (Bottom). On day 18, the cells were stained with Alizarin Red S. Macroscopic images (Middle) and staining intensities (OD_550_) are shown (Top). (**C**) Cells were cultured as in (**B**) and RNA was subjected to the real time-RT-PCR analysis on day 18. Relative mRNA levels are plotted. Magnification of the images are x 1. Values are means ± S.D. n = 3 (**A** and **B**) or 4 (**C**). *P < 0.05 and **P < 0.01, vs. the aHDF cultured in osteogenic medium alone. N.D., no significant difference between the indicated groups.

**Figure 2:**
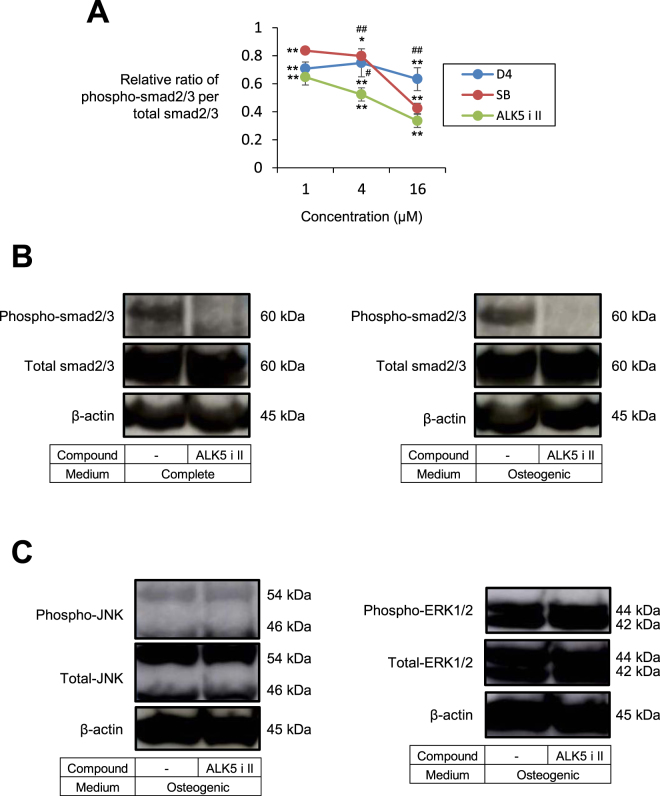
An addition of ALK5 i II inhibited smad2/3 signaling in fibroblasts. (**A**) HDFs were seeded onto 60-mm dishes, and on the next day culture supernatant was replaced by fresh complete medium with/without the indicated concentrations of TGF-β inhibitors. After culturing for 3 days, culture supernatant was replaced by fresh one, and cells were lysed 30 min later. ELISA was performed to evaluate relative ratio of phospho-smad2/3 per total smad2/3 (the value for untreated HDFs was set to 1). Values are means ± S.D. (n = 3). *P < 0.05, **P < 0.01 vs. untreated control. ^#^P < 0.05, ^##^P < 0.05 vs. HDFs treated with ALK5i II at the same concentration. (**B** and **C**) HDFs were seeded onto 60-mm dishes, and on the next day culture supernatant was replaced by fresh complete or osteogenic medium with/without ALK5 i II as indicated. After culturing for 3 days, culture supernatant was replaced by fresh one, and cells were lysed 30 min later. Western blotting analyses were performed using the indicated antibodies.

Some chemical compounds are known to enhance reprogramming of fibroblasts into iPS cells and to contribute to the maintenance of stem cell phenotypes, while others compounds promote differentiation of stem cells into osteoblast-like cells^12,15,24,25^. Among them, we selected twelve compounds and tested whether some of them may induce osteoblast-like phenotypes in HDFs. Human dermal fibroblasts (aHDFs) were cultured in osteogenic medium supplemented with each chemical compound for 28 days, and stained with Alizarin Red S to estimate calcium deposition (Supplementary Information Fig. S1). TGF-β R inhibitors D4476 (D4) and SB431542 (SB) induced relatively large amounts of calcium deposition, whereas other compounds failed to induce calcium deposition at higher levels than the osteogenic medium alone (Supplementary Information Fig. S2). Then we compared various TGF-β R inhibitors concerning their abilities to induce calcium deposition. The calcium phosphate was massively deposited after 21 days of treatment with the ALK5 inhibitor II (ALK5 i II), whereas D4 and SB induced considerable mineralization at day 28 but not at day 21 (Figs 1A and S2). Thus, the ALK5 i II was considered as the most potent compound for inducing osteoblastic conversion. Consistently, the ALK5 i II more strongly blocked the smad2/3 signaling than D4 and SB did (Fig. 2A). Therefore, we used ALK5 i II in the successive experiments.

aHDFs were cultured in osteogenic medium supplemented with ALK5 i II. The ALK5 i II treatment induced high ALP activity on day 13 (Fig. 1B, bottom). Alizarin Red S staining revealed that the calcified body occupied the entire surface of the culture well even at day 18 (Fig. 1B, middle). These phenotypes were quite similar to osteoblasts differentiated from mesenchymal stem cells (MSC-OBs). Quantitative measurement of the Alizarin Red S staining confirmed that the ALK5 i II-treated cells deposited as much calcium phosphate as the MSC-OBs did (Fig. 1B, top). The ALK5 i II treatment also induced expression of ALP, osterix and osteocalcin (OC) mRNA at comparable levels to those in MSC-OBs on day 18 (Fig. 1C).

We also examined whether ALK5 i II induced osteoblast-like phenotypes in fibroblasts of other origins. As a result, human dermal fibroblasts derived from other individuals (HDF45, HDF69 and HDF22) as well as human gingival fibroblasts showed a high ALP activity and deposited calcium phosphate after culturing in osteogenic medium supplemented with ALK5 i II (Supplementary Information Figs S3 and S4).

We analyzed TGF-β/smad signaling in aHDFs treated with ALK5 i II. Phosphorylated smad2/3 was detected in aHDFs cultured in the complete medium, whereas the smad2/3 was totally dephosphorylated by treatment with ALK5 i II (Fig. 2B, left). Similar results were also obtained using cells that were cultured in osteogenic medium with or without the ALK5 i II (Fig. 2B, right). The ERK1/2 and JNK were highly phosphorylated regardless of whether ALK5 i II was present or not (Fig. 2C). Data are consistent with previous reports that TGF-β R inhibitors such as ALK5 i II, SB and D4 do not affect ERK1/2 and JNK signaling^26–28^. The smad 7 feedback mechanism, which reportedly inhibits both TGF-β and BMP signaling^29^, was partially inhibited by the ALK5 i II treatment, while Smad 1/5/9 was highly phosphorylated with or without the ALK5 i II treatment (Supplementary Information Fig. S5). Therefore, inhibition of the smad2/3 signaling may be involved in the phenotypic conversion of the cells into osteoblasts induced by ALK5 i II.

### Cells treated with ALK5 i II and VitD3 exhibited further typical osteoblast phenotypes

**Figure 3:**
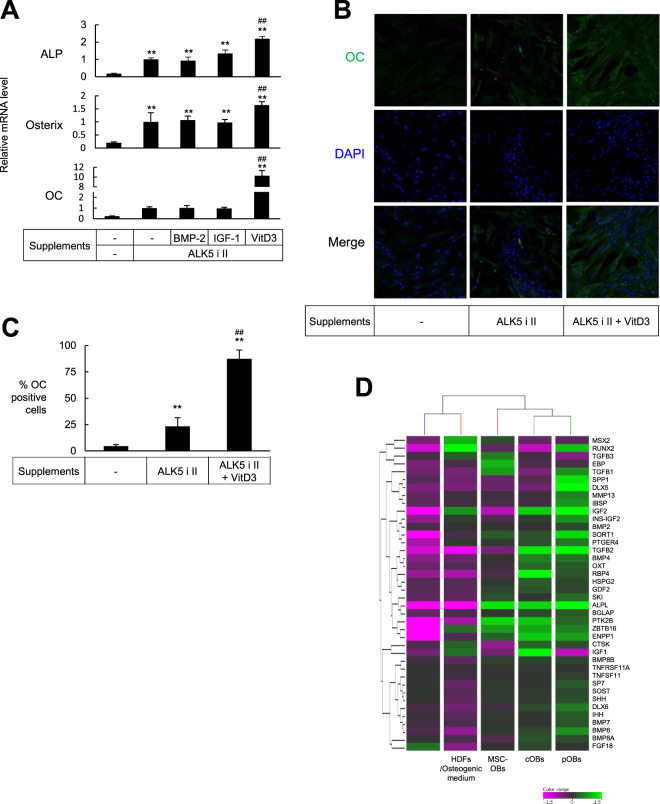
HDFs were efficiently converted into OC-producing osteoblasts by culturing with ALK5 i II and VitD3. (**A**) HDFs were cultured in osteogenic medium supplemented with ALK5 i II and the indicated supplements. After culturing for 18 days, real time RT-PCR was performed to evaluate mRNA for the indicated gene. Values are means ± S.D. n = 4. *P < 0.05 and **P < 0.01, v.s. HDFs cultured in osteogenic medium alone. ^#^*P* < 0.05 and ^##^*P* < 0.01, v.s. HDFs cultured in osteogenic medium supplemented with ALK5 i II. (**B** and **C**), HDFs were cultured in osteogenic medium supplemented with ALK5 i II and VitD3 for 18 days (cOBs). The cOBs, the HDFs cultured in osteogenic medium supplemented with ALK5 i II and the HDFs cultured in complete medium as control were stained with anti-OC antibody and DAPI. Fluorescence microscopic images (magnification was x200) (**B**) and proportion of OC producing cells (**C**) are shown. Values are means ± S.D. (n = 4). **P < 0.01. v.s. control. ^##^*P* < 0.01, v.s. HDFs cultured in osteogenic medium supplemented with ALK5 i II. (**D**) RNA was extracted from HDFs, HDFs cultured in the osteogenic medium for 18 days, cOBs induced as in (**B**), osteoblasts differentiated from MSCs (MSC-OBs), and pOBs. DNA microarray analysis was performed to evaluate mRNA for the genes encoding osteoblasts-related transcription factors, signaling proteins and soluble factors. Heat map and hierarchical clustering data are shown. The genes with increased expression are colored green, whereas those with decreased expression are colored pink as indicated in the color range. The expression level of each gene was normalized to median signal intensity.

In addition to ALK5 i II, we supplemented the osteogenic medium with BMP2, IGF-1 or VitD3, which were reported to directly enhance osteoblastic differentiation^30–32^. We found that VitD3 most remarkably elevated the expression of ALP, osterix and OC mRNA (Fig. 3A). The cells cultured in osteogenic medium supplemented with both ALK5 i II and VitD3 more abundantly formed calcified nodules (Supplementary Information Fig. S6) compared with cells cultured in osteogenic medium supplemented with ALK5 i II alone. Based on these findings, the cells treated with both ALK5 i II and VitD3 were brought to further detailed analyses as representative chemical compound-mediated directly converted osteoblasts (cOBs).

Immunostaining experiments indicated that the cOBs expressed OC at a high level, and showed that the efficiency of conversion of fibroblasts into osteoblasts was 87.3 ± 1.4% (Fig. 3B and C).

To estimate the similarity between cOBs and normal primary osteoblasts (pOBs), we examined the mRNA expression of the genes encoding osteoblasts-related transcription factors, signaling molecules, and soluble factors by DNA microarray analysis. The hierarchical clustering analysis revealed that the cOBs were more closely similar to pOBs than to fibroblasts, and the similarity between the cOBs and pOBs was higher than that between MSC-derived OBs and pOBs (as shown by the dendrogram in the Fig. 3D).

### cOBs contributed to bone repair after transplantation into mice at an artificial bone defect lesion

**Figure 4:**
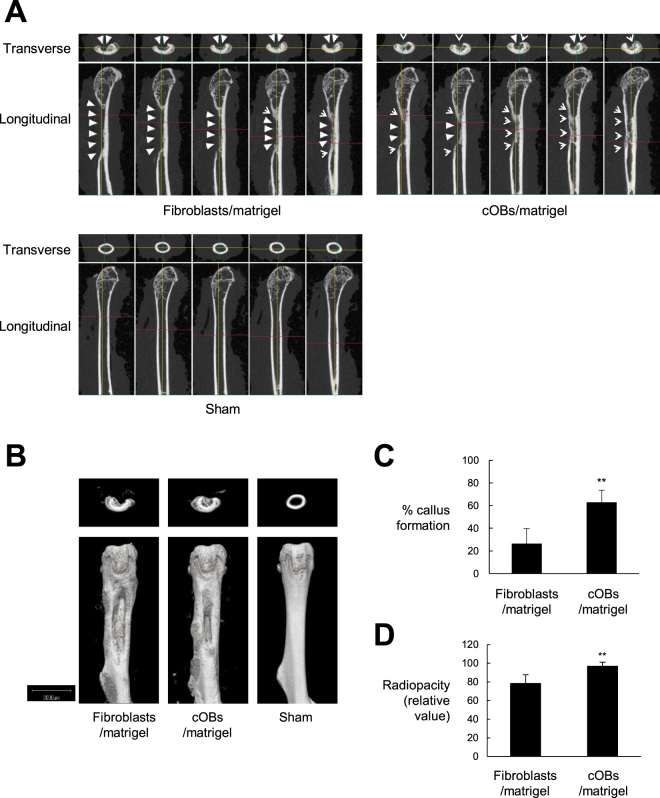
cOBs facilitated bone repair *in vivo*. HDFs were cultured in osteogenic medium supplemented with ALK5 i II and VitD3 for 13 days (cOBs). The cOBs and the HDFs cultured in complete medium as control were inoculated into artificial segmental bone defect lesion in femoral diaphysis in NOG mice. Bone defect was not created in the sham-operated mice. Twenty-one days later, mice were sacrificed and μCT imaging of the femur was performed. (**A** and **B**), Longitudinal and transverse serial 100 μm slice images (**A**) and 3D-constructed μCT images (**B**) of the femurs of a representative mouse are shown. White triangles indicate bone defect lesions, while arrow heads indicate regenerated bone tissue. (**C** and **D**), The means ± S.D. of the percentages of callus formation (**C**) and the relative radiopacity at the bone defect region (value for the sham operated group are set to 100%) (**D**) are plotted (n = 4). **P < 0.01.

To examine whether cOBs can enhance bone regeneration, we transplanted cOBs and fibroblasts as a control into the bone defect lesions that were surgically created at the femurs of immunodeficient NOG mice. Bone repair was estimated by radiographical and histological examinations 21 days after transplantation. Radiographically, the defect lesion in a cOB-transplanted femur was almost totally covered by regenerated callus, although complete bridging of the defect was not achieved at this point. The callus was only faintly formed in the femur grafted with fibroblasts (Fig. 4A and B). The cOB transplantation resulted in a significantly higher degree of callus formation compared with the transplantation with fibroblasts (Fig. 4C). Further, the radiopacity at the defective area was higher in the cOB-transplanted femur than in the fibroblast-transplanted femur, as shown by the μCT transmission images (Supplementary Information Fig. S7) and statistical analysis of them (Fig. 4D). Thus, the bone mass was elevated by the transplantation with cOBs.

**Figure 5:**
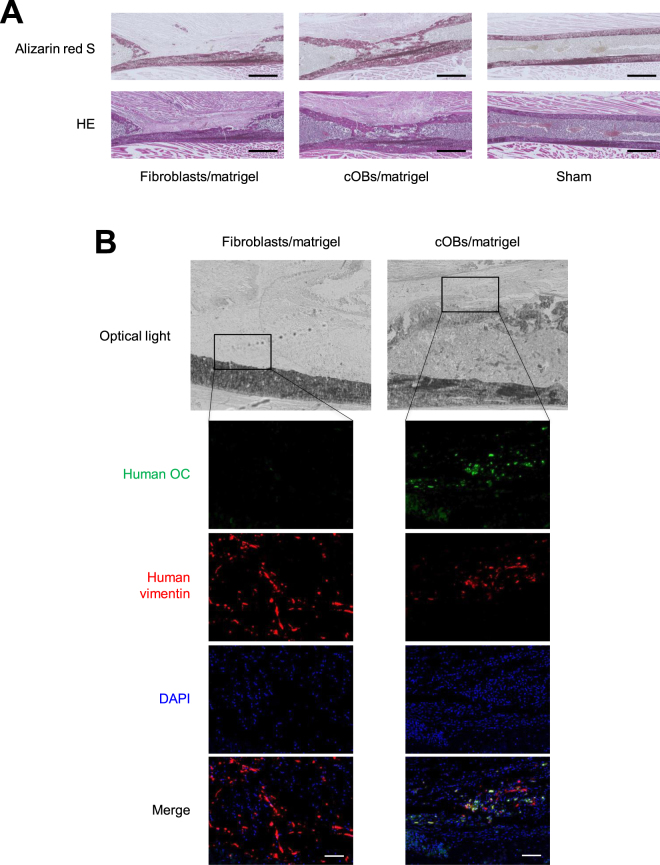
cOBs contributed to bone tissue regeneration *in vivo*. Transplantation experiment was performed as in Fig. 3. Twenty-one days after the cell inoculation, mice were sacrificed and the femur was excised. (**A**) Serial sections of the femur tissue were stained with Alizarin Red S (Upper) and H & E. (Lower). Scale bar = 1 mm. (**B**) Sections were stained with anti-human OC antibody followed by FITC-labeld secondary antibody, anti-human vimentin antibody followed by PE-labed secondary antibody, and DAPI. Optical light and fluorescence microscopic images are shown. Scale bar = 100 μm.

Histological analysis also showed massive callus formation (Fig. 5A, Lower) and extensive ossification of the callus (Fig. 5A Upper) at the bone defect lesion in the cOB-transplanted femur. In contrast, the callus was only partially formed in the femur transplanted with fibroblasts. These results indicated that transplantation of cOBs enhanced bone healing at the bone defective sites in NOG mouse femurs.

Finally, we tried to detect distribution of the transplanted cOBs and the bone matrix protein that they produced in the regenerated bone tissue. For these purposes, human OC and vimentin were visualized by immunohistochemical staining using species-specific antibodies. The human OC was detected at and around the regenerated callus in the cOB-transplanted femur, while human vimentin-positive cells were located on the surface of the callus and in the thickened periosteum (Fig. 5B).

These results strongly suggest that the donor cells produced bone matrix *in vivo*, which may have contributed to the promotion of bone healing.

## Discussion

It has been reported that inhibition of TGF-β R signaling enhanced reprogramming of somatic cells into iPS cells^12–14^. A TGF-β R inhibitor substituted for Sox2^18^ or Oct4^33^ in the induction of reprogramming and maintenance of pluripotency. An addition of a TGF-β R inhibitor increased 5- to 8- fold the efficiency of direct conversion of fibroblast into cardiomyocytes induced by transduction of some cardiomyocyte-specific transcription factor genes^34,35^. A TGF-β R inhibitor was also used as an essential component in the cocktails of small molecules that induced direct conversion of fibroblasts into neural cells^21,22^ and into cardiomyocytes^23^. In contrast to these previous studies, in which a TGF-β R inhibitor was used in combination with gene transfer and/or other chemical compounds, the TGF-β R inhibitor was basically the sole chemical compound necessary for cell fate conversion from fibroblasts into osteoblasts, although an addition of VitD3 further elevated expression levels of the osteoblast-specific genes.

In previous studies, SB was most frequently used as a TGF-β R inhibitor, but we found that ALK5 i II was more potent than SB in converting fibroblasts into osteoblasts. SB induced fibroblasts to exhibit osteoblast phenotypes to some extent, but the effect was remarkably weaker than that of the ALK5 i II (Fig. 1). Among the three ALK5 inhibitors i.e., D4, SB and ALK5 i II, the ALK5 i II most strongly inhibited smad2/3 signaling and induced osteoblastic conversion at the highest efficiency (Figs 1A and 2A and Supplementary Information Fig. S2). The ALK5 i II specifically inhibits ALK5, whereas SB inhibits ALK4, 5 and 7^26^. Meanwhile, D4 is known as a casein kinase inhibitor 1, while it specifically inhibits ALK5 among the TGF-β R family members^27^.

TGF-β is produced by osteoblasts and osteocytes, while its roles in osteogenesis are very much complicated. It was reported that TGF-β induced migration of MSCs and enhanced bone fracture healing^36^. Consistently, inhibition of TGF-β signal impaired bone fracture healing by suppressing recruitment and differentiation of immature osteoblasts^37^. In contrast, it has been reported that SB treatment elevated physiological differentiation of osteoblasts from MSCs, periodontal ligament cells and pre-osteoblasts that have intrinsic capabilities of osteogenic differentiation^38–40^. The TGF-β signal enhanced osteoblast differentiation at early stages, whereas it inhibited mineralization and proliferation of osteoblasts at later stages of differentiation^41^. Some other reports implied that TGF-β may either stimulate or inhibit differentiation of osteoblasts depending on their concentrations, compositions of culture medium, and differentiation stages and densities of the target cells^42,43^.

VitD3 enhanced differentiation and maturation of osteoblasts^44^. VitD3 positively regulated expression of some osteoblast-specific genes including OC and Runx2 that possessed copies of VitD responsive element (VDRE) sequences at their promoter regions^45^. Moreover, administrations of VitD prevented the progression of osteoporosis^46^.

The present procedure can possibly provide a feasible bone regeneration therapy against bone diseases, taking advantage of its high efficiency of conversion, of the high capability of cOBs to produce bone matrix and promote bone tissue regeneration, and of the fact that it does not necessitate exogenous gene transfer for conversion.

## Methods

### Cells

Normal human dermal fibroblasts derived from breast of 18-years-old male (aHDFs), Normal primary osteoblasts (pOBs) derived from a human femur and normal mesenchymal stem cells (MSCs) derived from a human adipose tissue were purchased from Lonza (Basel, Switzerland) and Kurabo (Kurashiki, Japan), respectively.

### Reagents and medium

Small molecular compounds are described in Supplemental Information Table S1. Bone morphogenetic protein-2 (BMP-2), and insulin-like growth factor-1 (IGF-1) were purchased from ReproTech (Rocky Hill, USA). 1α, 25-dihydroxy Vitamin D3 (VitD3) was purchased from Cayman (cat No. 71820). Dulbecco’s modified Eagle’s medium (DMEM) was supplemented with 100 mM non-essential amino acids, 100 U/mL penicillin, 100 μg/mL streptomycin and 10% fetal bovine serum (FBS) and used as the complete medium. Osteogenic medium consisted of the complete medium supplemented with 50 μg/mL ascorbic acid, 10 mM β-glycerol phosphate, and 100 nM dexamethasone.

### Cell culture

Fibroblasts were cultured in the complete medium, which was replaced by fresh one every 3 to 4 days. To convert fibroblasts into osteoblasts, fibroblasts were resuspended in the complete medium and seeded onto 60-mm dishes at a density of 1 × 10^5^ cells/dish (for DNA microarray analyses and transplantation experiments), 60-mm dishes at a density of 2 × 10^5^ cells/dish (for ELISA and western blotting), or 24-well plates at 1 × 10^4^ cells/well (for ALP staining, Alizarin red S staining, qRT-PCR and immunofluorescence) (day −1). On the next day, the culture medium was replaced by the osteogenic medium supplemented with small molecular compounds and/or cytokines. ALK5 i II was added to the medium at a concentration of 4 μM unless otherwise stated. In some experiments, BMP-2, IGF-1 or VitD3 was added to the medium at final concentrations of 100 ng/mL (BMP-2 and IGF-1) or 5 nM (VitD3). The culture medium was replaced with fresh one every 3 to 4 days. MSC-derived osteoblasts (MSC-OBs) were induced by culturing the MSCs in the osteogenic medium for 18 days.

### Cell staining

For Alizarin Red S staining, cells were fixed with 10% formaldehyde followed by staining with Alizarin Red S solution (Sigma Aldrich). For quantitative measurement of Alizarin Red S staining, the staining solution was harvested from the culture dish and optical density (OD) of each solution was measured at 550 nm. ALP staining was performed as previously described^47^.

### Real time RT-PCR

Total RNA was reverse-transcribed using ReverTra Ace qPCR RT Master Mix (Toyobo), and the resultant cDNA was mixed with Real-Time PCR Master Mix (Applied Biosystems, Waltham, MA, USA) and matching probes/primers specific for human β-actin, ALP, osterix or OC genes (Applied Bioscience; Hs00287164_m1, Hs01029144_m1, Hs01866874_s1, and Hs01587814_g1, respectively). Real time PCR was carried out on a Step One Plus Real-Time PCR System (Applied Biosystems). All values (average ± SD) were normalized with respect to the β-actin mRNA level in each sample, and relative values were calculated.

### ELISA

Cells were extracted in lysis buffer, and the cell lysates were subjected to ELISA using the PathScan^®^ Phospho Smad2/3 and PathScan^®^ Total Smad2/3 ELISA kit (Cell Signaling Technology) in accordance with manufacture’s instruction. The OD_450_ value (average ± SD) for phospho-smad2/3 was divided by that for total smad2/3 in each sample, and the ratio was normalized to the value for untreated HDFs (set to 1).

### Western blotting

Cells were extracted in lysis buffer containing a protease inhibitor cocktail and a phosphatase inhibitor cocktail (Nacalai Tesque) for 1 hour on ice. The lysates were centrifuged at 15,000 rpm for 15 minutes, heated at 70 °C for 10 min, and separated by SDS-PAGE (15 µg protein/lane). After transferred to a PVDF membrane using i-Blot 2 system (Life technology), the blot was probed overnight at 4 °C with the antibodies listed in the Supporting Information Table S2. After 1-hour incubation with HRP-labeled anti-rabbit or anti-mouse immunoglobulin antibody (GE Healthcare, Buckinghamshire, UK) (diluted at 1:20,000) at room temperature, signals were visualized using the ECL Select detection regent (GE Healthcare) and analyzed by Image Quant LAS 500 (GE Healthcare).

### Immunostaining

Cells were fixed in 4% paraformaldehyde at 4 °C for 30 min. After blocking, the cells were incubated with anti-human OC antibody (Bio-Rad) followed by another incubation with secondary antibody conjugated with Alexa fluor 488 (Thermo Fisher Scientific, Waltham, MA, USA). Cell nuclei were also stained with DAPI (Thermo Fisher Scientific). The cells were observed under a fluorescence microscope (BZ-X710; Keyence, Osaka, Japan). OC -positive and negative cells were counted by BZ-II Analyzer software (Keyence, Osaka, Japan) to calculate the percentage of OC -producing cells as follows: %OC producing cells = the number of OC (+) DAPI (+) cells per total number of DAPI (+) cells

### DNA microarray analysis

RNA was obtained from cells and subjected to DNA microarray analysis using the GeneChip Human Gene 1.0 ST Array (Affymetrix) as described previously^9^. Scanned data were analyzed using Expression Console (Affymetrix) and GeneSpring version 14.1 (Agilent Technologies). The microarray data have been deposited to GEO with the accession number GSE101140.

### Surgical procedure and cell transplantation

All animal experiments were approved by the Committee for Animal Research, Kyoto Prefectural University of Medicine, and all animal care was provided in accordance with institutional guidelines. Male NOG mice (Charles River) at 12-wk of age were anesthetized with isoflurane. A segmental bone defect approximately 6 mm in diameter was created at the diaphysis of the left femur using a dental drill under pouring water. HDFs and cOBs were resuspended in a 1:2 mixture of medium and Matrigel (BD Bioscience), and inoculated to the bone defect lesion at 5 × 10^5^ cells/mouse.

### Radiographic and histological assessment

Mice were euthanized with a lethal dose of isoflurane. Thighs were dissected, fixed with 10% neutral-buffered formalin, and subjected to μCT imaging (Scan Xmate-L090; Com Scan Techno). For volumetric quantification, longitudinal sections through the segmental defect were analyzed using ImageJ software to calculate percentage of callus formation. After radiologic assessment, explants were embedded in SCEM compound (Leica Microsystems) and quick-frozen. Frozen specimens were sectioned into 6-μm slices and subjected to H&E and Alizarin Red S staining, then fixed further with 4% paraformaldehyde and blocked with Blocking One Histo (Nacalai Tesque) and goat anti-mouse IgG (Abcom) before immunostaining with mouse anti-human OC (Bio-Rad) and rabbit anti-human vimentin (Abcam) antibodies and DAPI. For visualization of osteocalcin and vimentin, cryosections were incubated with Alexa Fluor 488-conjugated anti-mouse IgG (Invitrogen) and Alexa Fluor 546-conjugated anti-rabbit IgG (Invitrogen) antibodies, respectively. Fluorescence and optical light microscopic images were obtained and analyzed with BZ-X710 and BZ-II Analyzer software (Keyence).

### Data analysis

Data are expressed as mean ± standard deviation (S.D.). Statistical significance was analyzed using Student’s t-test and ANOVA with Tukey-Kramer post hoc test. P < 0.05 was considered significant.

### Accession numbers

The microarray data have been deposited to GEO with the accession number GSE101140.

## Electronic supplementary material

Supporting information
