# Supplementary material for: Direct phenotypic conversion of human fibroblasts into functional osteoblasts triggered by a blockade of the transforming growth factor-β signal
Source: Sci Rep. 2018 May 31;8:8463. doi: 10.1038/s41598-018-26745-2 (PMC5981640; doi:10.1038/s41598-018-26745-2)
Supplement: Supplementary file 1 — Supporting information [file 41598_2018_26745_MOESM1_ESM.pdf]

Supporting information

Direct phenotypic conversion of human fibroblasts into functional osteoblasts triggered by a blockade of the transforming growth factor-β signal

Kenta Yamamoto,[1, 2] Tsunao Kishida,[1] Kei Nakai,[1, 2] Yoshiki Sato,[2] Shin-ichiro Kotani,[1] Yuta Nishizawa,[1] Toshiro Yamamoto,[2] Narisato Kanamura,[2] & Osam Mazda [1,*]

[1]Department of Immunology, Kyoto Prefectural University of Medicine, Kamigyo, Kyoto 602-8566, Japan

[2]Department of Dental Medicine, Kyoto Prefectural University of Medicine, Kamigyo, Kyoto 602-8566, Japan

[*]Corresponding author: Osam Mazda at the Department of Immunology, Kyoto Prefectural University of Medicine, Kamigyo, Kyoto 602-8566, Japan.

Supporting Information Materials and Methods

Cells

Normal human dermal fibroblasts derived from abdomen of 45-years-old Hispanic female (HDF45), 22-years-old black female (HDF22), and temple of 69-years-old Caucasian female (HDF69) were purchased from Toyobo Life Science (Osaka, Japan). Normal human gingival fibroblasts were obtained from the dissociated gingiva specimen of a healthy donor (22-years-old Asian male) who provided informed consent. The study was approved by the institutional ethics committee.

Cell staining

For staining by the von Kossa's reaction, the cells were fixed with 10% formalin and incubated with freshly prepared 5% silver nitrate solution (ScyTek laboratories, Logan, UT, USA) for 30 min with exposure to UV light. After washing with distilled water, the cells were treated with 5% sodium thiosulfate solution (ScyTek laboratories).

Nomenclature (Abbriviation)	Molecular Weight	Final concentration	Source (Provider, Catalogue number)
I-BET151 (I-BET)	415.4	2 μM	Sigma, SML0666
Pifthrin-α (PF)	367.3	2 μM	Wako, 166-23131
PD0325901 (PD)	482.2	2 μM	Wako, 162-25291
2-Methyl-5-hydroxytryptamine hydrochloride (2-Me-5HT)	226.7	2 μM	TOCRIS, 0558
CX4945 (CX)	349.8	2 μM	BioVision, 2459-5
CHIR99021 (CHIR)	465.3	2 μM	Cayman, 13122
Forskolin (FSK)	410.5	2 μM	Wako, 067-02191
3-Deazaneplanocin A Hydrochloride (DZnep)	262.3	50 nM	Cayman, 13828
Pitavastatin (PiS)	881.0	100 nM	Cayman, 15414
Simvastatin (SS)	418.6	100 nM	Sigma, S6169
D4476 (D4)	398.4	1, 2, 4 μM	Calbiochem, 218705
SB431542 (SB)	550.5	1, 2, 4 μM	Wako, 192-16542
LY2157229 (LY21)	369.4	1, 4 μM	Cayman, 15312
LY364947 (LY36)	272.3	1, 4 μM	Cayman, 13341
SD208 (SD)	352.8	1, 4 μM	TOCRIS, 3269
ALK5 inhibitor II (ALK5 i II)	287.3	1, 4 μM	StemRD, ALK-010

Supporting Information Table S1

List of chemical compounds used in this study.

Antibodies	Species	Dilution	Provider
Anti-Smad2/3	Rabbit	1/1,000	Cell Signaling Technology (Danvers, MA, USA)
Anti-phospho Smad2/3	Rabbit	1/1,000	Cell Signaling Technology (Danvers, MA, USA)
Anti-Smad 1	Rabbit	1/1,000	Cell Signaling Technology (Danvers, MA, USA)
Anti-phospho-Smad1/5/9	Rabbit	1/1,000	Cell Signaling Technology (Danvers, MA, USA)
Anti-ERK1/2	Rabbit	1/1,000	Cell Signaling Technology (Danvers, MA, USA)
Anti-phospho-ERK1/2	Rabbit	1/1,000	Cell Signaling Technology (Danvers, MA, USA)
Anti-JNK	Rabbit	1/1,000	Cell Signaling Technology (Danvers, MA, USA)
Anti-phospho-JNK	Rabbit	1/1,000	Cell Signaling Technology (Danvers, MA, USA)
Anti-β-actin	Rabbit	1/1,000	Cell Signaling Technology (Danvers, MA, USA)
Anti-Smad7	Mouse	1/200	Santa Cruz Biotechnology (Dallas, TX, USA)

Supporting Information Table S2

List of first antibodies used in the western blot analysis in this study.

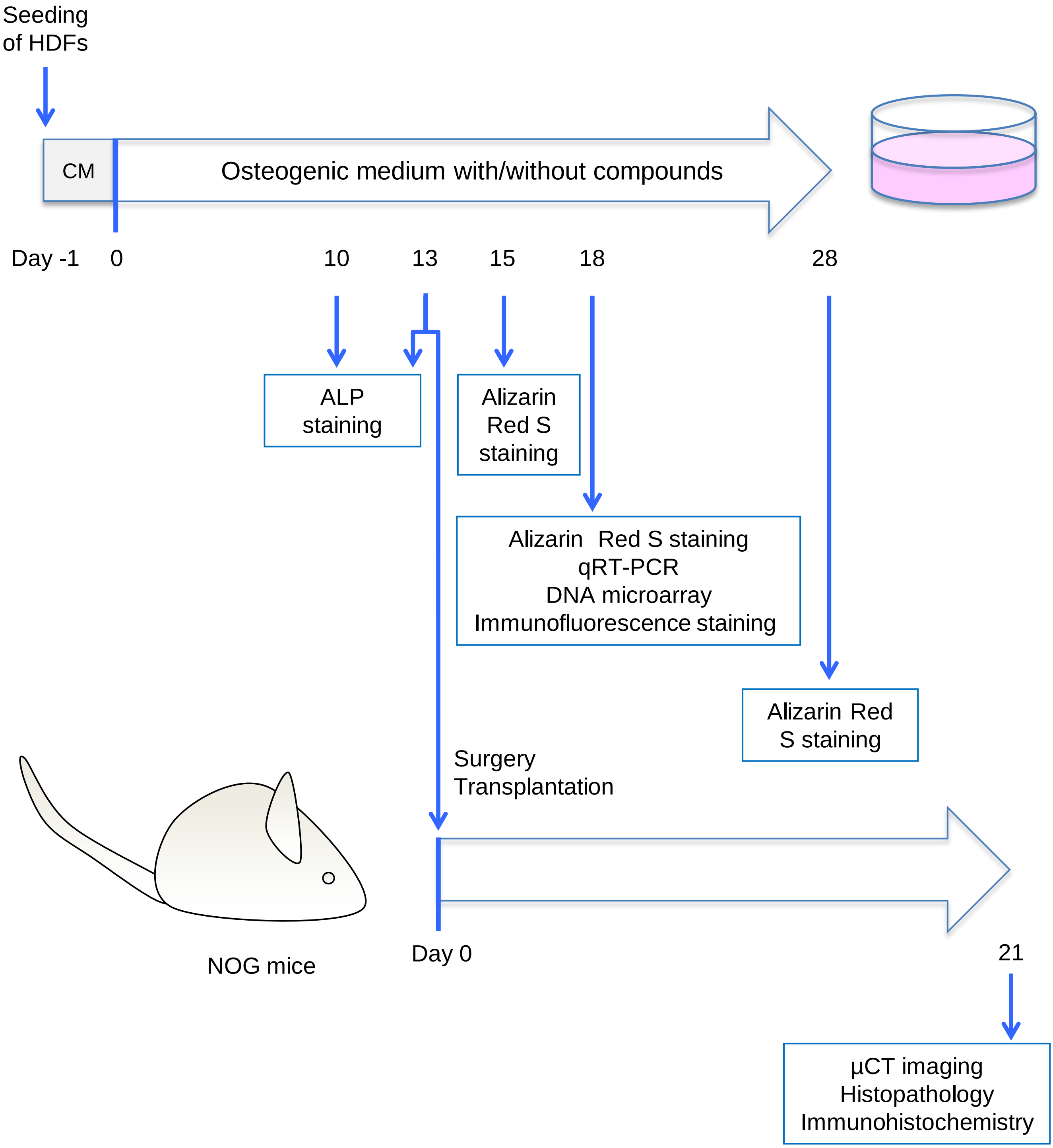

Supporting Information Fig. S1
Scheme of the experimental design. CM: compete medium.

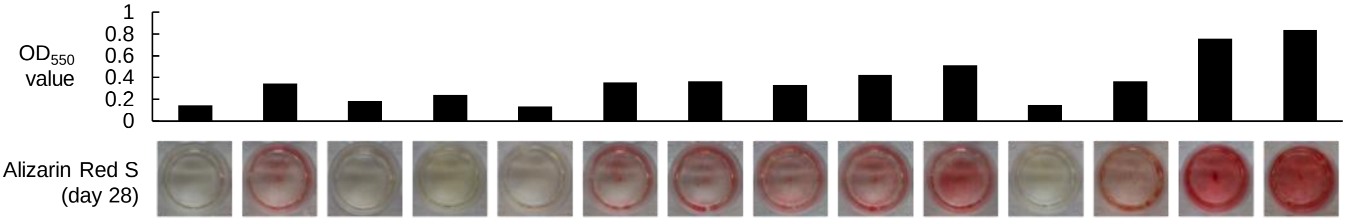

Compounds	-	-	I-BET	PF	PD	2-Me-5HT	CX	CHIR	FSK	DZnep	PiS	SS	D4	SB
Medium	Complete	Osteogenic												

Supporting Information Fig. S2

Fibroblasts were induced to deposit calcium phosphate by the TGF-β R inhibitors. Human dermal fibroblasts (aHDFs) were seeded in 24-well plates and cultured in complete medium or osteogenic medium supplemented with the indicated chemical compounds. Twenty-eight days later, the cells were stained with Alizarin Red S. Macroscopic images (original magnification: x 1) (Lower) and staining intensities (OD_{550}) (Upper) are shown. n=1. Representative of 2 independent experiments.

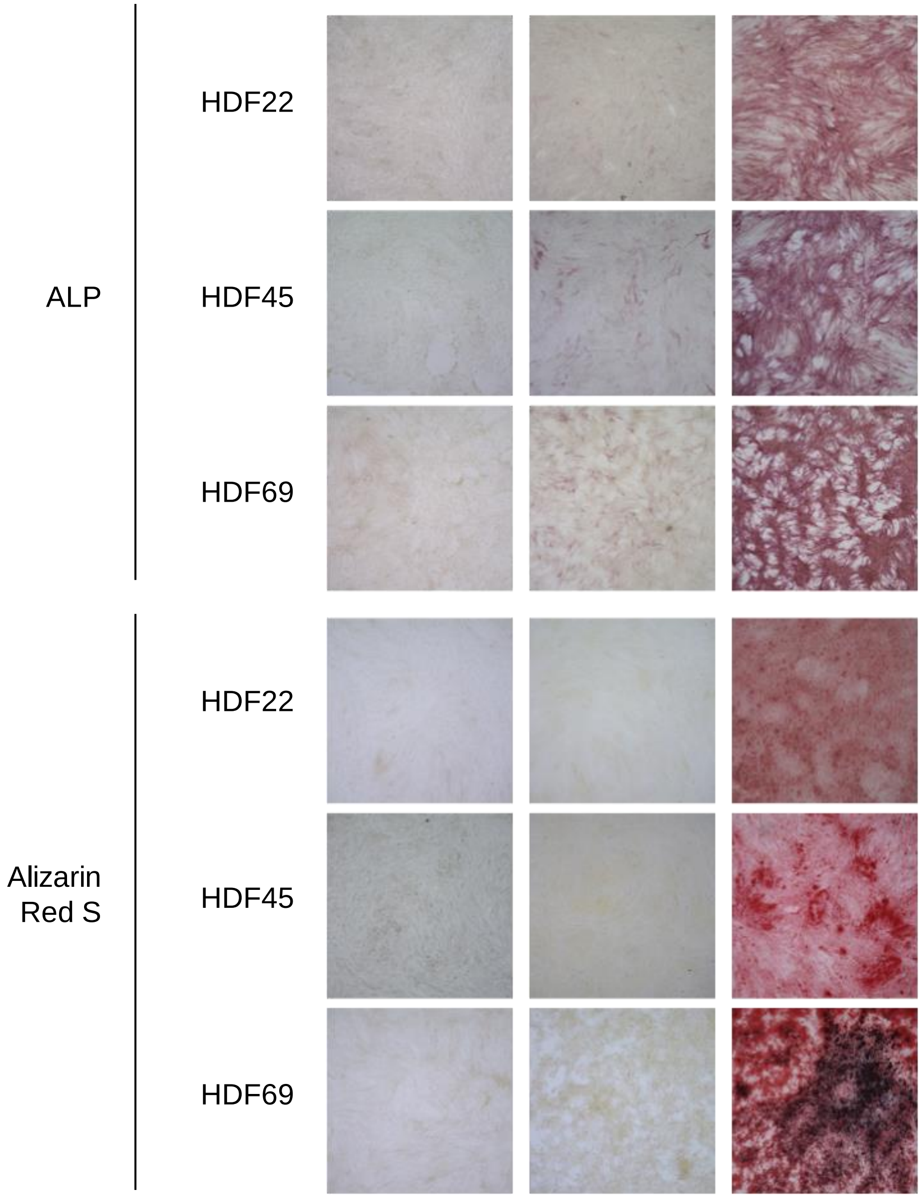

Compound	-	-	ALK5 i II
Medium	Complete	Osteogenic	

Supporting Information Fig. S3

ALK5 i II treatment induced osteoblast-like phenotypes in fibroblasts derived from dermis of various individuals. Human dermal fibroblasts from three different individuals (HDF45, HDF22, and HDF69) were seeded in 24-well plates and cultured in complete medium, osteogenic medium or osteogenic medium supplemented with ALK5 i II. Ten and 15 days later, cells were subjected to ALP staining and Alizarin Red S staining, respectively. Microscopic images are shown (original magnification was x 40).

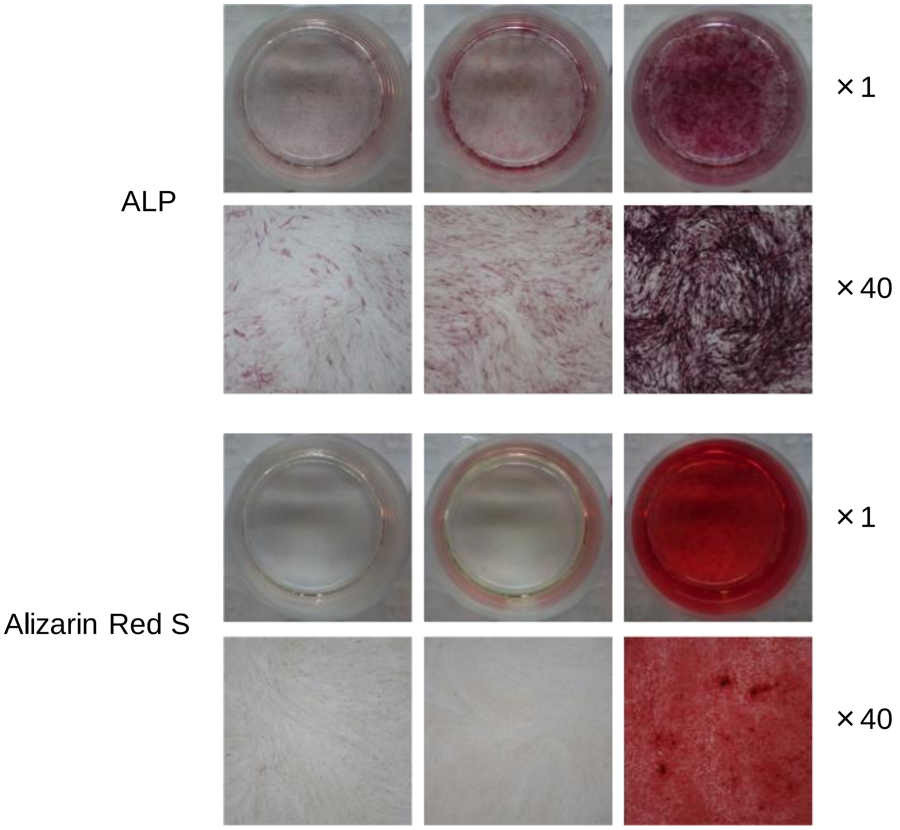

Compound	-	-	ALK5 i II
Medium	Complete	Osteogenic	

Supporting Information Fig. S4

ALK5 i II treatment induced osteoblast-like phenotypes in human gingival fibroblasts. Human gingival fibroblasts were seeded in 24-well plates and cultured in the indicated medium as in the Supplementary Fig. S2. Thirteen or 18 days later, cells were subjected to ALP staining (Upper) and Alizarin Red S staining (Lower), respectively. Macroscopic and microscopic images are shown (original magnifications were x 1 and x 40 as indicated).

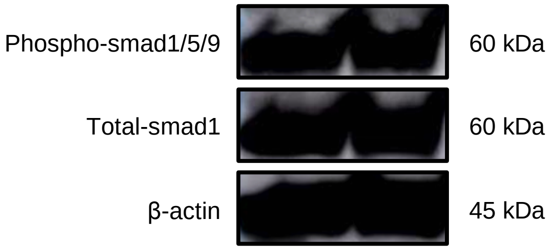

Compound	-	ALK5 i II
Medium	Osteogenic	

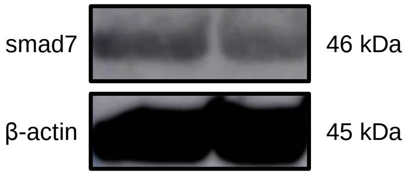

Compound	-	ALK5 i II
Medium	Osteogenic	

Supporting Information Fig. S5

ALK5 i II didn’t influence smad 1/5/9 signaling, while partially inhibiting smad7 expression. HDFs were seeded onto 60-mm dishes, and on the next day culture supernatant was replaced by fresh osteogenic medium with/without ALK5 i II. After culturing for 3 days, culture supernatant was replaced by fresh one, and cells were lysed 30 min later. Western blotting analyses were performed using the indicated antibodies.

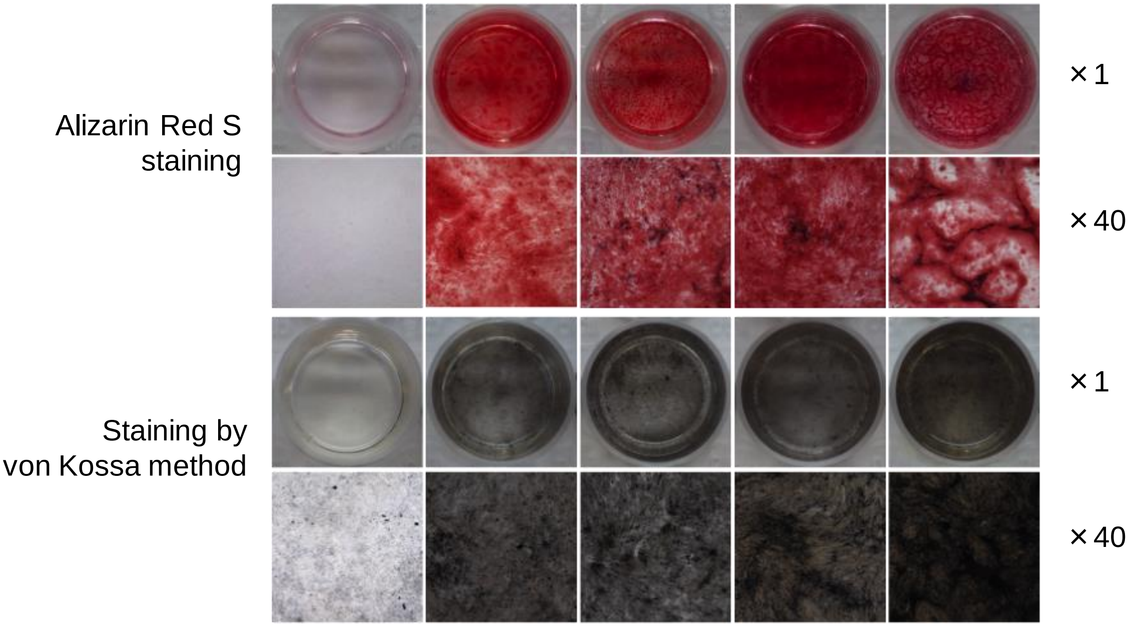

Supplements	-	-	BMP-2	IGF-1	VitD3
Compound	-	ALK5 i II			

Supporting Information Fig. S6
HDFs were cultured in osteogenic medium supplemented with ALK5 i II and the indicated supplements for 18 days. Cells were then subjected to Alizarin Red S staining (Upper) or staining by the von Kossa method (Lower). Macroscopic and microscopic images are shown (original magnifications were x 1 and x 40 as indicated).

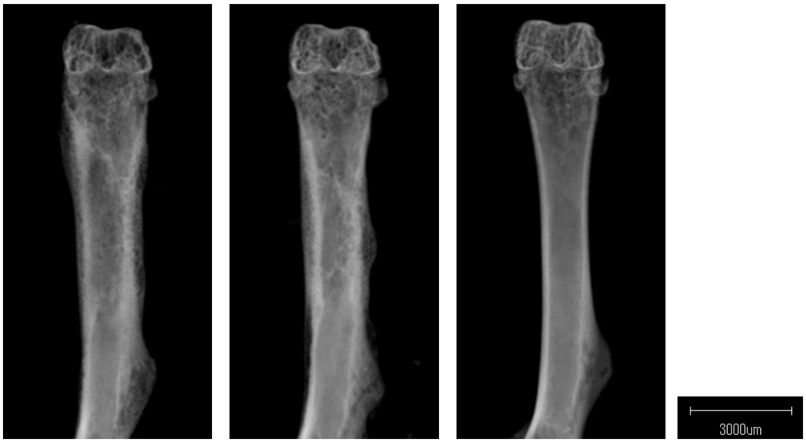

Supporting Information Fig. S7

Transplantation of cOBs markedly enhanced healing of bone defect and increased bone mass at the bone defect lesions. Transplantation experiment and μCT imaging were performed as in Fig. 4. (A) μCT transmission images of the mouse femur are shown.

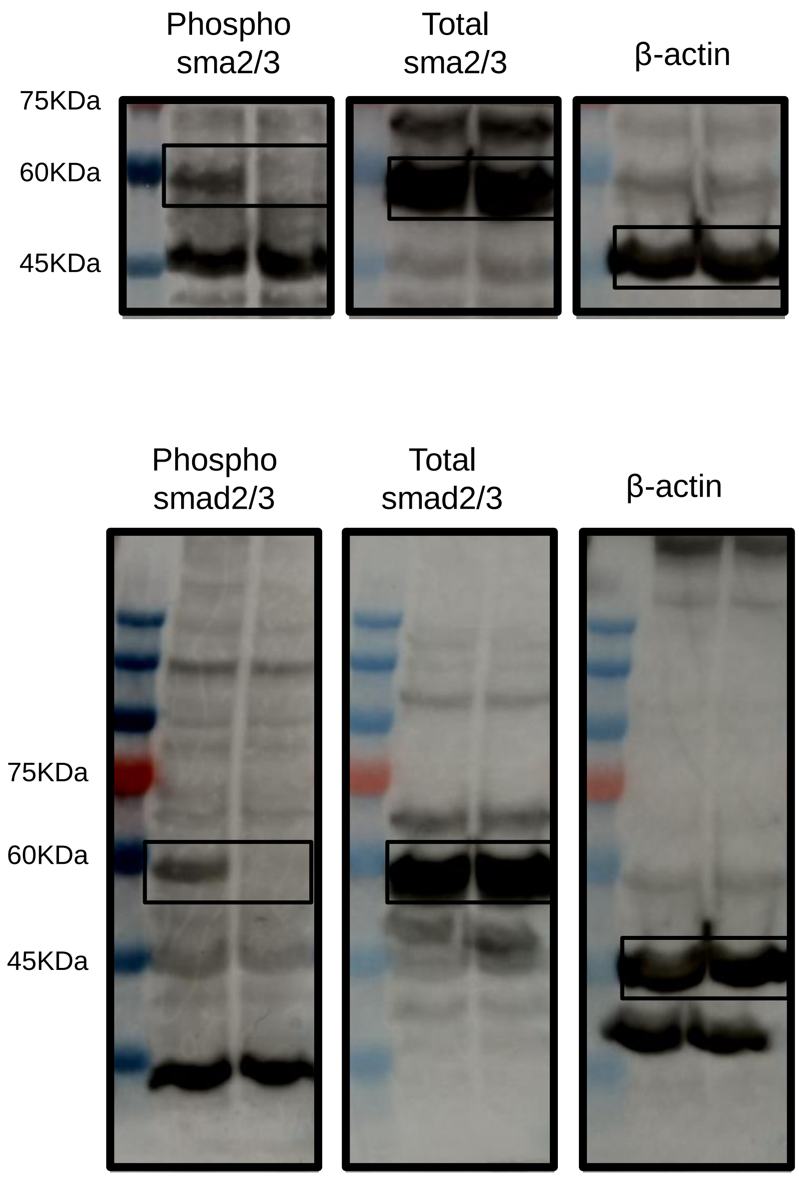

Supporting Information Fig. S8

Original unedited western blot images of Fig. 2B. Framed regions indicated the utilized regions for Fig. 2B.

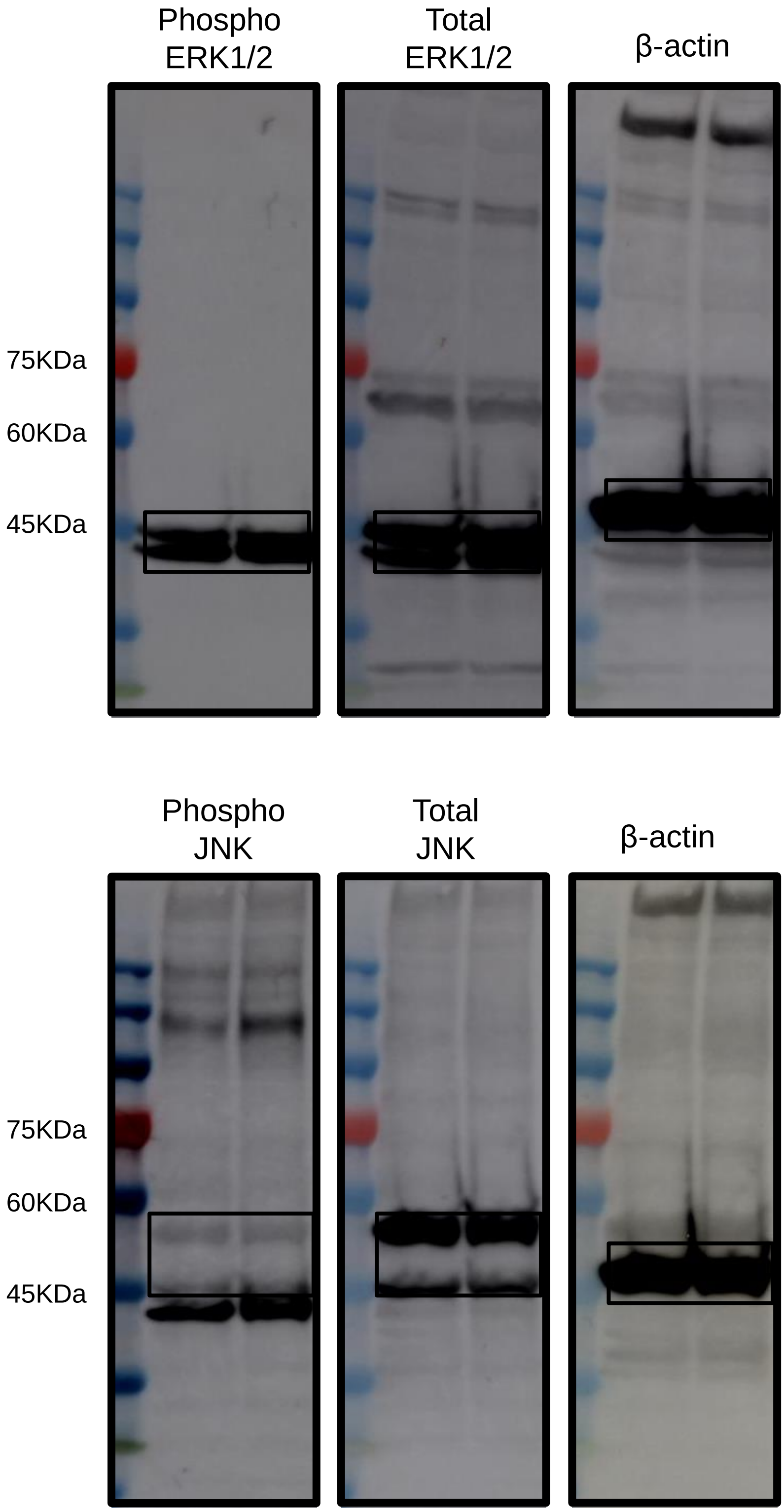

Supporting Information Fig. S9
Original unedited western blot images of Fig. 2C. Framed regions indicated the utilized regions for Fig. 2C.

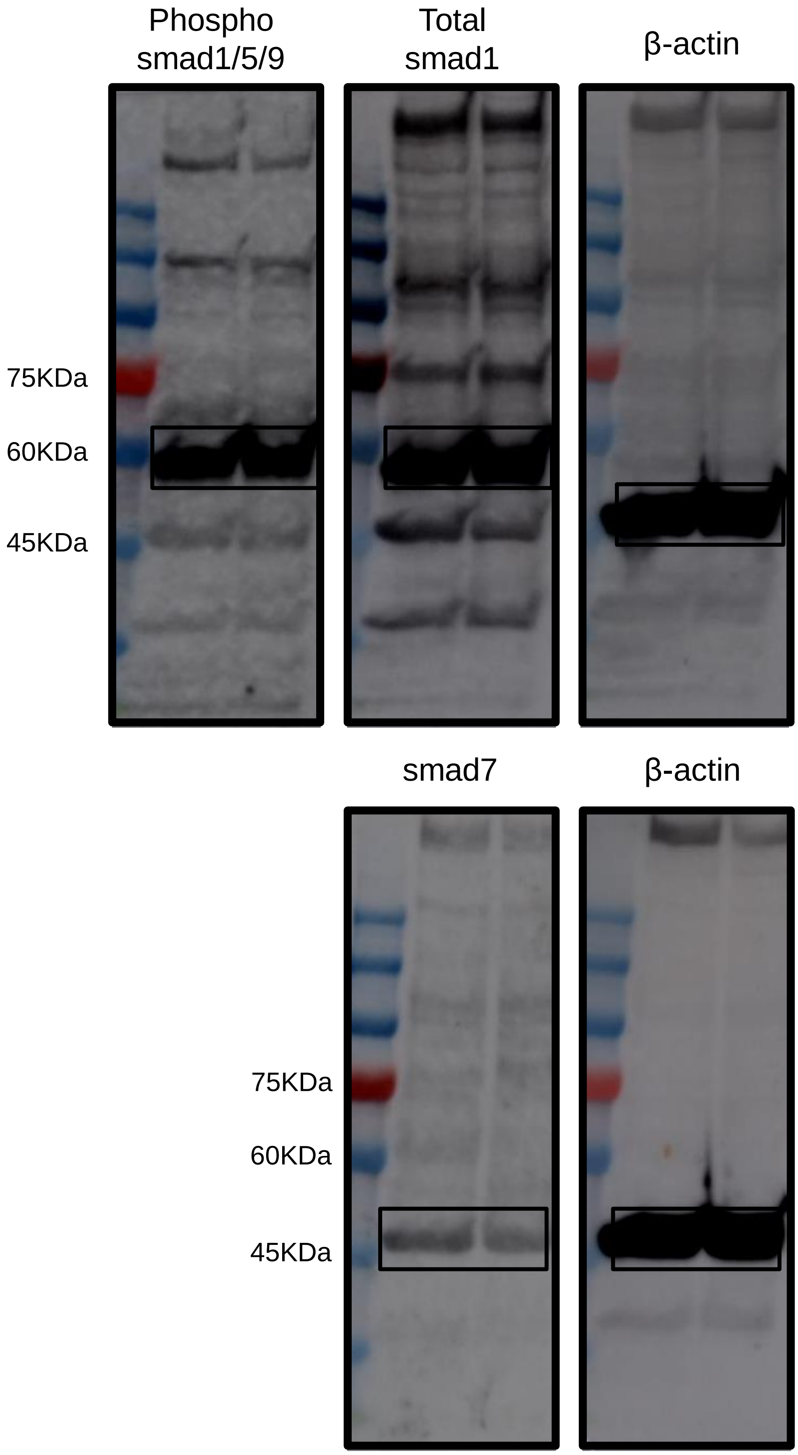

Fig. S10
Original unedited western blot images of Supplementary Information Fig. S5. Framed regions indicated the utilized regions for Supplementary Information Fig. S5.
